# Flux Crystal Growth, Crystal Structure, and Optical Properties of New Germanate Garnet Ce_2_CaMg_2_Ge_3_O_12_

**DOI:** 10.3389/fchem.2020.00091

**Published:** 2020-02-18

**Authors:** Jie Chen, Hong Yan, Akihide Kuwabara, Mark D. Smith, Yuki Iwasa, Hiraku Ogino, Yoshitaka Matsushita, Yoshihiro Tsujimoto, Kazunari Yamaura, Hans-Conrad zur Loye

**Affiliations:** ^1^Research Center for Functional Materials, National Institute for Materials Science, Tsukuba, Japan; ^2^Graduate School of Chemical Sciences and Engineering, Hokkaido University, Sapporo, Japan; ^3^Nanostructures Research Laboratory, Japan Fine Ceramic Center, Nagoya, Japan; ^4^Department of Chemistry and Biochemistry, University of South Carolina, Columbia, SC, United States; ^5^Electronics and Photonics Research Institute, National Institute of Advance Industrial Science and Technology, Tsukuba, Japan; ^6^Materials Analysis Station, National Institute for Materials Science, Tsukuba, Japan

**Keywords:** flux crystal growth, garnet, germanate, single crystal, photoluminescence

## Abstract

A new germanate garnet compound, Ce_2_CaMg_2_Ge_3_O_12_, was synthesized via flux crystal growth. Truncated spherical, reddish-orange single crystals with a typical size of 0.1–0.3 mm were grown out of a BaCl_2_-CaCl_2_ melt. The single crystals were characterized by single-crystal X-ray diffraction analysis, which revealed that it adopted a cubic garnet-type structure with *a* = 12.5487(3) Å in the space group *Ia*−3*d*. Its composition is best described as *A*_3_*B*_2_*C*_3_O_12_, where Ce/Ca, Mg, and Ge occupied the *A, B*, and *C* sites, respectively. A UV–vis absorption spectroscopy measurement on the germanate garnet revealed a clear absorption edge corresponding to a band gap of 2.21 eV (λ = 561 nm). First-principle calculations indicated that the valence band maximum was composed of Ce 4*f* bands, whereas the conduction band minimum mainly consisted of Ce 5*d* bands. These findings explain the observed absorption edge through the Ce 4*f* → 5*d* absorption. Photoluminescence emission spectra exhibited a very broad peak centered at 600 nm, corresponding to transition from the lowest energy *d* level to the 4*f* levels.

## Introduction

**Figure 1:**
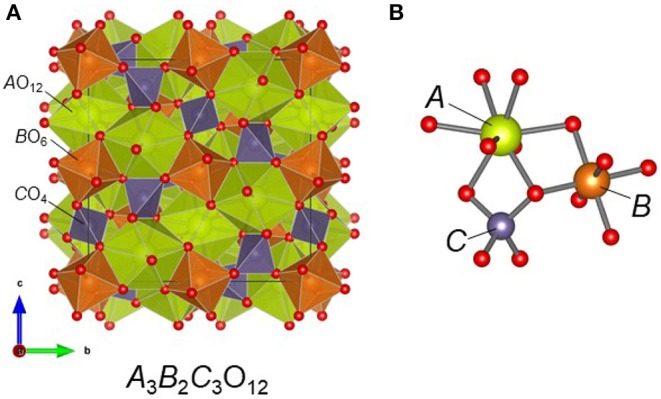
**(A)** Crystal structure of the garnet compound *A*_3_*B*_2_*C*_3_O_12_ and **(B)** the local coordination environment around the metal cations. In Ce_2_CaMg_2_Ge_3_O_12_, Ce/Ca, Mg, and Ge atoms occupy the A, B, and C sites, respectively.

The garnet structure, having the general chemical formula {*A*}_3_[*B*]_2_(*C*)_3_O_12_, has been widely studied as a host material for various optical applications, such as laser amplifiers, color converters, scintillators, and cathode ray phosphors (Zhiguo and Meijerink, 2017). In particular, the Ce^3+^-doped Y_3_Al_5_O_12_ garnet phosphor (YAG:Ce) is one of the most interesting materials in terms of practical application as a blue-to-yellow converter in white-emitting diodes. Although YAG:Ce exhibits good thermal and chemical stability and high luminescence efficiency, improvements to the low thermal quenching temperature and cool correlated color temperature remain significant issues (Bachmann et al., 2009; Shao et al., 2011; Ueda et al., 2015). In principle, the 5*d*−4*f* emission bands in Ce^3+^-doped phosphors are strongly influenced by the host lattice through crystal field splitting of the 5d levels of the Ce^3+^ ion. In the garnet host, there are three types of cation sites: the {*A*} site with 8-fold dodecahedral coordination, the [*B*] site with 6-fold octahedral coordination, and the (*C*) site with 4-fold tetrahedral coordination (Figure 1). The *A* site is typically occupied by rare-earth (*RE*) ions such as La^3+^, Gd^3+^, or Lu^3+^, as well as by Y^3+^, and by alkaline earth ions such as Ca^2+^. The *B* site is occupied by smaller ions that prefer octahedral coordination environments, such as Mg^2+^ Mn^3+^, Fe^3+^, Sc^3+^, Al^3+^, or Zr^4+^, while the *C* site accommodates ions that take on tetrahedral coordination, including Al^3+^, Ga^3+^, Si^4+^, or Ge^4+^ ions. The dodecahedral site, which the trivalent Ce^3+^ ion prefers to occupy, connects to the adjacent *A, B*, and *C* sites through common oxygen atoms via corner and edge sharing. Thus, the crystal field impinging on the Ce^3+^ ions is created not only by the *A* site cations but also the *B* and *C* site cations (Setlur et al., 2006; Muñoz-García and Seijo, 2010; Muñoz-García et al., 2010). Owing to the wide range of cations that can be accommodated by the garnet structure, new compositions of garnet phosphors that compensate for the above-mentioned shortcomings of YAG:Ce have been successfully synthesized.

When considering the crystal chemistry of the garnet family, the ability of the cation sites, especially the *A* site, to accommodate different elements, is an important factor (Geller, 1967). A large number of garnet compounds have been previously reported (Tauber et al., 1958; Reinen, 1964; Lévy and Barbier, 1999; Zhiguo and Meijerink, 2017) but the variety of *RE* ions in the *A* site is typically limited to *RE* = Gd–Lu and Y, all smaller than the desirable Eu^3+^ cation, because incorporation of larger cation causes markedly unfavorable lattice distortions around the dodecahedral sites. To the best of our knowledge, only very few garnet compositions that include early *RE* ions larger than Gd^3+^ have been synthesized (via various techniques such as the sol-gel method and hydrothermal reaction), and include: Eu_3_Al_5_O_12_ (Garskaite et al., 2007), *RE*_3_Te_2_Li_3_O_12_ (*RE* = Pr–Eu) (Kasper, 1969), *RE*_3_W_2_Li_3_O_12_ (*RE* = Pr, Nd) (Kasper, 1969), *RE*_3_Fe_5_O_12_ (*RE* = Pr–Eu) (Dukhovskaya et al., 1973; Komori et al., 2009a,b; Guo et al., 2011), La_3_Sc_2_Ga_3_O_12_ (Malysa et al., 2018), *RE*_3_Ga_5_O_12_ (*RE* = Pr–Eu) (Sawada, 1997; Sawada et al., 2016), Li_7_La_3_Zr_2_O_12_ (Awaka et al., 2009), and Li_5_La_3_Sb_2_O_12_ (Murugan et al., 2008). It is notable that even among these, achieving a garnet composition with Ce^3+^ fully occupying the *A* site is challenging; however, Ce^3+^ doping as high as 56 at.% with respect to Y^3+^ has been achieved in YFe_5_O_12_ via the glycothermal process (Rongjin et al., 2013).

In this study, we report the flux crystal growth of the new germanate oxide {Ce_2_Ca}[Mg]_2_(Ge)_3_O_12_, which crystalizes in the garnet structure in the space group *Ia*−3*d* with *a* = 12.5487(3) Å. The garnet phase was synthesized via the flux crystal growth method where truncated spherical, reddish-orange single crystals were obtained from a BaCl_2_−CaCl_2_ melt. High-temperature solid state reactions failed to yield the target phase, even as a polycrystalline powder, suggesting that the phase is metastable. Herein we discuss the crystal structure, electronic structure, and optical properties of Ce_2_CaMg_2_Ge_3_O_12_.

## Experimental

### Crystal Growth

**Figure 2:**
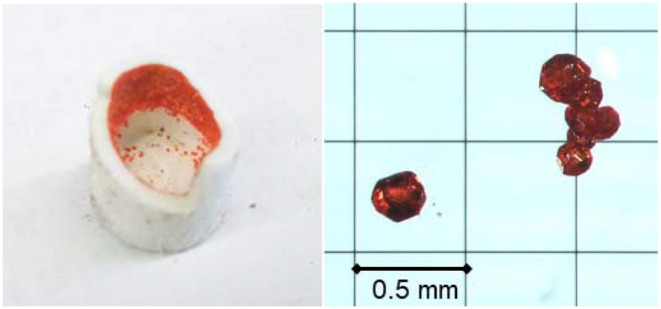
Photographs of single crystals of Ce_2_CaMg_2_Ge_3_O_12_ grown on the inner wall of a MgO crucible.

Single crystals of Ce_2_CaMg_2_Ge_3_O_12_ were grown via the flux method using a eutectic BaCl_2_−CaCl_2_ mixture (Bugaris and zur Loye, 2012). For Ce_2_CaMg_2_Ge_3_O_12_, a magnesia crucible was loaded with 1 mmol CeO_2_ (Aldrich, 4N), 1 mmol of GeO_2_ (Rare Metallic, 4N), 1 mmol of S (High Purity Materials, 4N), 3.1 mmol of BaCl_2_ (Rare Metallic, 3N), and 3.1 mmol of CaCl_2_ (Rare Metallic, 3N). The top of the crucible was closed with a magnesia cap, and the crucible was sealed inside a silica tube under vacuum. As described later, the magnesia crucible was found to act as a magnesium source. The starting materials were heated in a box furnace to 900°C at 150°C/h, held for 25 h, cooled to 500°C at 5°C/h, and then allowed to cool naturally to room temperature. The products were washed in distilled water, aided by sonication, before the reddish-orange transparent truncated spherical crystals of Ce_2_CaMg_2_Ge_3_O_12_, together with pale-purple transparent crystals of CeOCl, were collected via vacuum filtration. The typical dimensions of the single crystals of the garnet compound were 0.3 × 0.3 × 0.3 mm^3^ (Figure 2). The structure of Ce_2_CaMg_2_Ge_3_O_12_ was determined by single-crystal X-ray diffraction analysis.

Ce_2_CaMg_2_Ge_3_O_12_ single crystals were obtained unintentionally because our original target system was germanium-containing oxysulfide. As described later, sulfur was not incorporated in the garnet structure, and no oxysulfide compounds could be obtained.

### Single Crystal Structure Analysis

X-ray intensity data from an orange polyhedron were collected at 301(2) K using a Bruker D8 QUEST diffractometer equipped with a PHOTON 100 CMOS area detector and an Incoatec microfocus source (Mo Kα radiation, λ = 0.71073 Å) (Bruker AXS Inc., 2016). The data collection covered 100% of the reciprocal space to 2θ_max_ = 75.2°, with an average reflection redundancy of 35.3 and *R*_int_ = 0.064 after absorption correction. The raw area detector data frames were reduced and corrected for absorption effects using the SAINT+ and SADABS programs (Krause et al., 2015; Bruker AXS Inc., 2016). Final unit cell parameters were determined by least-squares refinement of 3812 reflections taken from the data set. An initial structural model was obtained with SHELXT (Sheldrick, 2015). Subsequent difference Fourier calculations and full-matrix least-squares refinement against *F*^2^ were performed with SHELXL-2018 using the ShelXle interface (Hübschle et al., 2011).

### Solid State Synthesis

The synthesis of polycrystalline powder samples of Ce_2_CaMg_2_Ge_3_O_12_ was attempted using CeO_2_, CaCO_3_ (or CaO), MgO, and GeO_2_ in a stoichiometric ratio. The mixture was ground intimately, pelletized, and heated in a flowing N_2_ or H_2_ (20%)–Ar (80%) mixed gas atmosphere or in an evacuated sealed tube using a tubular furnace at temperatures ranging from 900 to 1,300°C.

### XRD, UV-vis, PL, PLE, and Magnetic Measurements

Single crystals of Ce_2_CaMg_2_Ge_3_O_12_ were crushed with an agate mortar and pestle to obtain fine powders used for obtaining synchrotron X-ray powder diffraction (SXRD) patterns, UV–vis diffuse reflectance spectra, and photoluminescence (PL) and photoluminescence excitation (PLE) spectra. The products obtained via solid state reactions were examined at room temperature by powder XRD analysis using a Rigaku MiniFlex X-ray diffractometer (Cu Kα radiation) in the 2θ range of 5–65° with a step size of 0.04°. SXRD measurement was performed at room temperature using a one-dimensional detector installed on BL15XU, NIMS beamline at SPring-8 in Japan. The synchrotron radiation X-rays were monochromatized to a wavelength of 0.65298 Å. The Ce_2_CaMg_2_Ge_3_O_12_ powder sample was loaded into a 0.1-mm diameter glass capillary. The diffraction data were recorded in 0.003° increments over the range 2–60° and analyzed by Rietveld refinement using the program RIETAN-FP (Izumi and Momma, 2007). Diffuse reflectivity measurements were performed at room temperature using a Shimadzu UV-2600 spectrophotometer equipped with an ISR-2600Plus integration sphere. The diffuse reflectance data were internally converted to absorbance by the instrument using the Kubelka–Munk function. The PLE and emission spectra were recorded using a fluorescence spectrophotometer (Hitachi F-7000). The magnetic susceptibility of Ce_2_CaMg_2_Ge_3_O_12_ was measured using a SQUID magnetometer (Quantum Design, MPMS-XL). The crushed single crystals were measured at an applied magnetic field (*H*) of 1 kOe in the range of 10–300 K under both zero-field-cooled (ZFC) and field-cooled (FC) conditions.

### First-Principles Calculations

First-principles total energy calculations of Ce_2_CaMg_2_Ge_3_O_12_ were performed using the projector augmented wave method (Blöchl, 1994; Kresse and Joubert, 1999) as implemented in the Vienna Ab-initio Simulation Package (VASP) (Kresse and Hafner, 1993; Kresse and Furthmüller, 1996a,b). In the present study, the cut-off energy for the plane wave basis was 550 eV. The exchange-correlation interaction potentials of electrons were handled within a framework of the generalized gradient approximation (GGA) of with the PBEsol type (Perdew et al., 2008). The configurations of the valence electrons of Ce, Ca, Mg, Ge and O were 5*s*^2^ 5*p*^6^ 4*f* ^1^ 5*d*^1^ 6*s*^2^, 3*s*^2^ 3*p*^6^ 4*s*^2^, 2*p*^6^ 3*s*^2^, 3*d*^10^ 4*s*^2^ 4*p*^2^, and 2*s*^2^ 2*p*^4^, respectively. Spin-polarized calculations were carried out. For Ce ions, the effect of the strong correlation interaction of the 4f orbital was treated based on the GGA+*U* method (Dudarev et al., 1998). The value of *U* was set to be 5.4 eV in this study (Jiang et al., 2009, 2012). Structure optimization calculations were carried out until the residual forces were <0.02 eV/Å.

## Results and Discussion

### Crystal Growth and Structure Determination

After washing the products inside the magnesia crucible with water to remove the solidified flux, we found that reddish-orange single crystals had grown on the inner wall of the tube (Figure 2) alongside with a plate-like pale-purple crystalline CeOCl byproduct. The EDS analysis of the reddish-orange crystals revealed the presence of Ce, Ca, Mg, and Ge in approximate atomic ratios of 1.9:1.0:2.1:2.6. The origin of the magnesium is the magnesia crucible that, apparently, was slightly dissolved by the flux during the reaction. Single-crystal X-ray diffraction analysis revealed that the product crystallized in the cubic system with *a* = 12.5479(4) Å. The space group *Ia*−3*d* (space group no. 230) was uniquely determined by the pattern of systematic absences in the intensity data and confirmed by structure solution. The product exhibits a garnet-type structure, wherein the asymmetric unit consists of one mixed Ce/Ca atomic site (Wyckoff site 24*c*), one Ge site (24*d*), one Mg site (16*a*), and one O site (96*h*). The composition of site 24*c* was determined by trial refinements of several models incorporating cationic elements determined by EDS to be present in the crystals (i.e., only Ce, Ca, Mg, and Ge). Modeling the site with mixed Ce/Ca occupancy resulted in the most reasonable model and is consistent with their similar ionic radii (${r_{\text{Ce}}}^{3+}$ = 1.143 Å, ${r_{\text{Ca}}}^{2+}$ = 0.97 Å) (Shannon, 1976) and their observed bond distances to O [2.427(4) and 2.547(4) Å, respectively]. To maintain overall charge balance, the Ce and Ca occupancies were fixed at 2/3 Ce and 1/3 Ca. Trial refinements with Ce and Ca occupancies constrained to sum to 1.0 but otherwise free to vary, refined closely to these values, supporting the decision to fix the occupancies at 2/3 Ce and 1/3 Ca. All atoms were refined with anisotropic displacement parameters. The final refined chemical composition was Ce_2_CaMg_2_Ge_3_O_12_, which is consistent with the result of the EDS analysis. The final *R*_1_ and *wR*_2_ converged to reasonable values of 3.83 and 5.51%, respectively. The goodness-of-fit value was 1.29. The incorporation of Ce^3+^ ions into the structure was consistent with the reddish-orange sample color. Details of the structure refinement are listed in Table 1. Atomic coordinates and atomic displacement parameters are listed in Table 2 and Supplementary Table 1, respectively. Selected bond distances and bond angles are compiled in Table 3.

**Table 1:** Results of structural refinement of Ce_2_CaMg_2_Ge_3_O_12_ using single-crystal XRD data.

Crystal dimensions (mm^3^)	0.080 7 × 0.050 × 0.030
Crystal system	Cubic
Space group	*Ia-3d* (#230)
*a* (Å)	12.5487 (3)
*V* (Å^3^)	1976.04 (14)
*Z*	8
ρ_cal_ (g/cm^3^)	5.235
Temperature (K)	301(2)
θ range (^°^)	3.978–37.590
μ (mm^−1^)	18.765
Crystal dimensions (mm^3^)	0.080 × 0.050 × 0.030
Collected reflections	17,162
Unique reflections	445
*R*_int_	0.0645
GOF	1.286
*R*_l_ for *F*o^2^ > 2σ(*F*o^2^)	0.0383
*wR*_2_ for *F*o^2^ > 2σ(*F*o^2^)	0.0551
Δρ_max_/Δρ_min_ (e/Å^3^)	0.931/−1.069

**Table 2:** Atomic coordinates and equivalent isotropic displacement parameters *U*_eq_ for Ce_2_CaMg_2_Ge_3_O_12_ obtained from the structure refinement using single-crystal XRD data.

**Atom**	**Site**	***x***	***y***	***z***	***Occp*.**	***U*_**eq**_ (Å^**2**^ × 10^**2**^)**
Ce^a^	24*c*	1/8	0	1/4	0.667	0.737 (15)
Ca^a^	24*c*	1/8	0	1/4	0.333	0.737
Mg	16*a*	0	0	0	1	0.75 (5)
Ge	24*d*	3/8	0	1/4	1	0.0660 (18)
O	96h	0.0948 (2)	0.1976 (3)	0.2852 (3)	1	0.68 (5)

a *For Ce and Ca atoms occupying the same site (24c), their site occupancies were fixed at 0.667 and 0.333, respectively, so that the sum of them was equal to unity. The U_eq_ values as well as the atomic coordinates were also constrained to the same values*.

**Table 3:** Selected interatomic distances and bond angles of Ce_2_CaMg_2_Ge_3_O_12_ at 301 K.

	**Bond distance (Å)**		**Bond angle (deg)**
Ce/Ca–O × 4	2.427 (4)	Ce/Ca–O–Mg	97.48 (12)
Ce/Ca –O × 4	2.547 (4)	Ce/Ca–O–Mg	101.26 (15)
Mg–O × 6	2.102 (3)	Ce/Ca –O–Ge × 2	95.60 (13)
Ge–O × 4	1.766 (3)	Ce/Ca–O–Ce/Ca × 2	101.13 (14)

**Figure 3:**
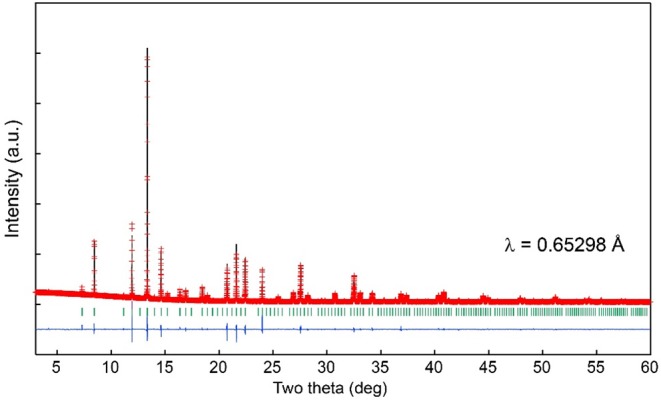
Observed (crosses), calculated (upper solid line), and difference (lower solid line) plots obtained from the Rietveld analysis of the room temperature synchrotron X-ray powder diffraction data collected using ground single crystals of Ce_2_CaMg_2_Ge_3_O_12_. Vertical lines represent expected Bragg peak positions.

Figure 3 shows the room-temperature synchrotron X-ray diffraction pattern collected from a powder sample obtained by finely grinding hand-picked single crystals. The model determined by the single-crystal structure analysis was used for the Rietveld refinement. The calculated pattern well-reproduced the observed pattern as the fitting converged smoothly with reasonable reliability indexes, *R*_wp_ = 5.37, *R*_B_ = 3.45, and *R*_F_ = 2.92. The final refined crystallographic data, including the atomic coordinates and isotropic displacement parameters are listed in Supplementary Table 2. The results are consistent with the results obtained from the single-crystal structure analysis.

### Solid State Reaction

Synthesis of a polycrystalline sample of Ce_2_CaMg_2_Ge_3_O_12_ was attempted by solid-state reactions using a stoichiometric mixture of CeO_2_, CaCO_3_ (or CaO), MgO, and GeO_2_. The reactions were carried out under vacuum, with mixed H_2_(20%)-Ar(80%) gas or N_2_ gas atmospheres at temperatures between 900 and 1,300°C. Unfortunately, none of the reaction conditions we examined yielded the target phase; but a substantial amount of unreacted CeO_2_ always remained in the products (see Supplementary Figure 1). A garnet structure was obtained as a minor phase at 1,300°C in the N_2_ gas atmosphere; however, the lattice parameter of the garnet phase was smaller by 0.5% compared with that for Ce_2_CaMg_2_Ge_3_O_12_ and the product was dark grayish-green. Therefore, if Ce atoms were incorporated into the lattice, the garnet phase obtained by solid state reaction should have a lower Ce concentration than that of Ce_2_CaMg_2_Ge_3_O_12_. Further heating at the same temperature after regrinding and pelletizing resulted in a partial decomposition of the garnet phase and an increase in the amount of CeO_2_, suggesting that the garnet phase was metastable under these reaction conditions.

### Stability of the Garnet Structure

As described earlier, the garnet structure can accommodate a wide range of elements in the three different cation sites, but the underlying stability of the garnet structure, including its tolerance for *RE* ions, is not yet well-understood. Our present germanate garnet exhibited an unusual occupancy of two-thirds of the *A* sites by Ce^3+^ ions, a Ce^3+^ concentration substantially higher than the 56 at.% Ce^3+^-doping concentration found in Y_1−x_Ce_*x*_Fe_5_O_12_ (*x* = 1.7) (Rongjin et al., 2013). Very recently, Song et al. have formulated the tolerance factor (τ) of the garnet structure (Song et al., 2019), which is analogous to the Goldschmidt tolerance factor describing the relationship of the chemical compositions and structural stability in perovskites (Goldschmidt, 1926). The τ of the garnet structure is expressed as

(1) $$\begin{array}{r} \tau =\frac{3\sqrt{{\left(r_{B}+r_{O}\right)}^{2}+\frac{4}{9}{\left(r_{A}+r_{O}\right)}^{2}\;}}{2\left(r_{C}+r_{O}\right)} \end{array}$$

where *r*_*A*_, *r*_*B*_, *r*_*C*_, and *r*_O_ represent the ionic radii of the *A, B, C* site cations and O^2−^ ion, respectively. The tolerance factor calculated for more than 100 garnet compounds falls within the range of 0.75–1.33. For the formula *RE*_3_*B*_2_*C*_3_O_12_. (*RE* = La–Lu, Y; *B* = *C*= Fe, Al, Ga), the τ values systematically increase toward unity with decreasing size of the *RE* ions, e.g., 0.76–0.93 from La to Lu for RE_3_Al_5_O_12_ and 0.89–1.02 for *RE*_3_Fe_5_O_12_) (Song et al., 2019). This is consistent with the general trend observed for their structural stability when containing *RE* ions. The formula *RE*_2_CaMg_2_Ge_3_O_12_ (*RE* = La–Lu, Y), including hypothetical compositions, exhibits a similar size dependence of the tolerance factor, but the τ values range from 1.06 for La, through 1.07 for Ce, to 1.15 for Lu. The stabilization of Ce_2_CaMg_2_Ge_3_O_12_ with a τ value close to unity seems to be compatible with the geometric requirements for the garnet structure. However, a favorable tolerance factor does not assure the success of the target phase formation via chemical synthesis. In fact, the solid-state reactions we examined to obtain Ce_2_CaMg_2_Ge_3_O_12_ were not successful. At present, the reason for the large amount of Ce ions incorporated into the garnet lattice is unclear; however, it is likely that the molten salts used in this study play a crucial role in stabilizing the phase under the flux reaction conditions. From the PXRD data of the products obtained by solid state reactions, it is apparent that CeO_2_ was not fully consumed in the reactions, indicating its low reactivity and slow atomic diffusion even at high temperatures. In the flux reaction, the BaCl_2_−CaCl_2_ salt likely dissolves CeO_2_ powder at a relatively low temperature, where the fact that the starting materials are now in solution is expected to decrease considerably the activation energy for reaction between the starting materials and thus yield the target garnet phase. We surmise that the Ca-Cl melt at high temperatures under vacuum acts as a reducing agent for Ce ions, likely forming Cl_2_. The formation of Ce^3+^ in the halide melt favors the stabilization of Ce_2_CaMg_2_Ge_3_O_12_ as well as of the byproduct CeOCl. Sulfur, which was a starting material for the flux reaction, was not found to significantly contribute to either the reduction of Ce ions nor to the formation of Ce_2_CaMg_2_Ge_3_O_12_. Performing the flux crystal growth in the absence of sulfur results in the same mixed product formation.

### Optical and Magnetic Properties

**Figure 4:**
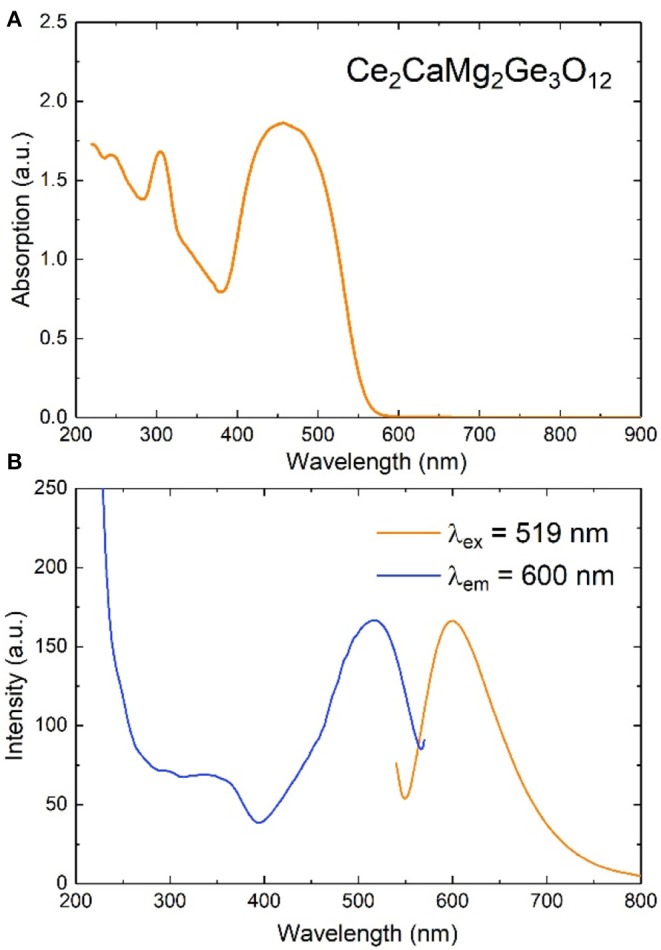
**(A)** UV–vis absorption spectrum, and **(B)** photoluminescence emission (λ_ex_ = 519 nm) and excitation (λ_em_ = 600 nm) spectra for Ce_2_CaMg_2_Ge_3_O_12_, collected at room temperature.

Figure 4A shows the UV–vis absorption spectrum collected for Ce_2_CaMg_2_Ge_3_O_12_, exhibiting a clear absorption edge at around 560 nm. An extrapolation of the linear portion of the absorption curve to the *x*-axis indicates an optical band gap of *E*_g_ = 2.22 eV. This steep increase in the absorption is followed by two broad sub-bands centered at 458 and 305 nm, also observed in the UV–vis absorption curves of YAG:Ce. These two absorption peaks can be assigned to the optical transitions from the Ce 4*f* ground state to the lowest and second-lowest excited states of the Ce 5*d* orbitals (5*d*_1_ and 5*d*_2_, respectively) (Bachmann et al., 2009). A third weak peak at around 250 nm is probably due to defects or impurities. The lowest absorption is in the blue spectral region, which results in the reddish orange color of the garnet compound. The photoluminescence emission (PE) and excitation (PLE) spectra of Ce_2_CaMg_2_Ge_3_O_12_ are shown in Figure 4B. The PE spectrum excited at 519 nm contains a broad band centered around 600 nm, which could be assigned to the transition from the 5*d*_1_ level to the two 4*f* levels split by spin-orbit coupling into ^2^F_5/2_ and ^2^F_7/2_. The maximum value of the emission band for Ce_2_CaMg_2_Ge_3_O_12_ is red-shifted compared to that of Y_2_Mg_3_Ge_3_O_12_:Ce(2%) (Jiang et al., 2010) but comparable to that for Gd_2_Mg_3_Ge_3_O_12_:Ce(2%) (Wu et al., 2007).

**Figure 5:**
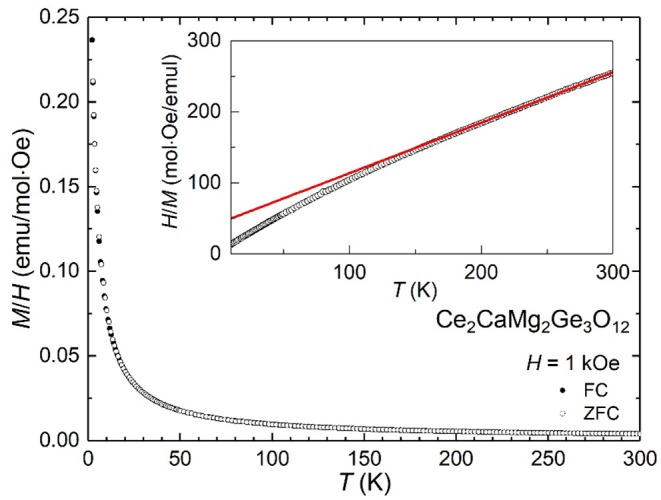
Magnetic susceptibility of Ce_2_CaMg_2_Ge_3_O_12_, measured in a magnetic field of 1 kOe. The inset shows its inverse χ vs. *T* plot. The red solid line is the fit to the Curie-Weiss law.

Figure 5 shows the temperature evolution of the magnetic susceptibility χ (= *M*/*H*) measured in a magnetic field *H* = 1 kOe. Both the ZFC and FC data increase smoothly with decreasing temperature, indicative of a paramagnetic state persisting down to low temperatures. No significant difference between the ZFC and FC data was observed in the temperature range between 10 and 300 K. Fitting χ(*T*) to the Curie–Weiss law yields *C* = 1.407(4) (emu K/mol) and θ = −59.9(9) K, where *C* and θ stand for the Curie and Weiss constants, respectively. The C value is somewhat smaller than the theoretical value expected from two mol Ce^3+^ ions with ^2^F_5/2_ per formula unit. The negative θ value suggests that Ce^3+^ ions are antiferromagnetically coupled to each other. The absence of a long-range magnetic order is probably due to a random distribution of Ce and Ca atoms on the 24*c* site.

### Theoretical Calculations

From the experimental crystal structure analysis of Ce_2_CaMg_2_Ge_3_O_12_, Ce, and Ca ions are found to occupy the *A* site of the *A*_3_*B*_2_*C*_3_O_12_ garnet structure. In first principles calculations using structure models under periodic boundary conditions, mixed occupancy of atomic sites cannot be directly computed. Therefore, we initially determined the preferred distribution of Ce and Ca ions on the *A* site with a ratio of 2:1 in a fixed size model having the garnet structure. We chose a primitive unit cell of the garnet structure as a base model. Structure models having symmetrically non-equivalent configurations of Ce and Ca ions were constructed. In total, 20 independent configurations of Ce and Ca ions on the *A*-site were found from the base model using the CLUPAN code (Seko et al., 2009). The mesh size of ***k***-point sampling was 3 × 3 × 3 in the Brillouin zone of the input structure models. We compared the total energies of these models obtained by structure optimization calculations.

**Figure 6:**
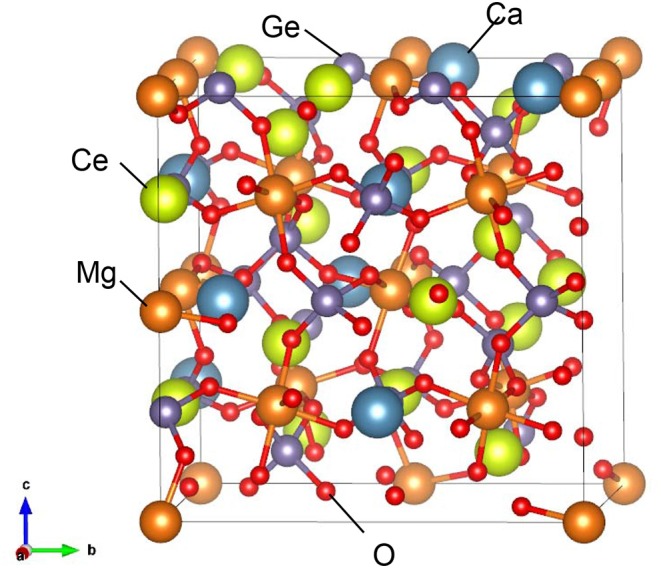
Most stable configurations of Ce_2_CaMg_2_Ge_3_O_12_ found by a series of first principles calculations in the present study. Search conditions are described in the main text.

**Figure 7:**
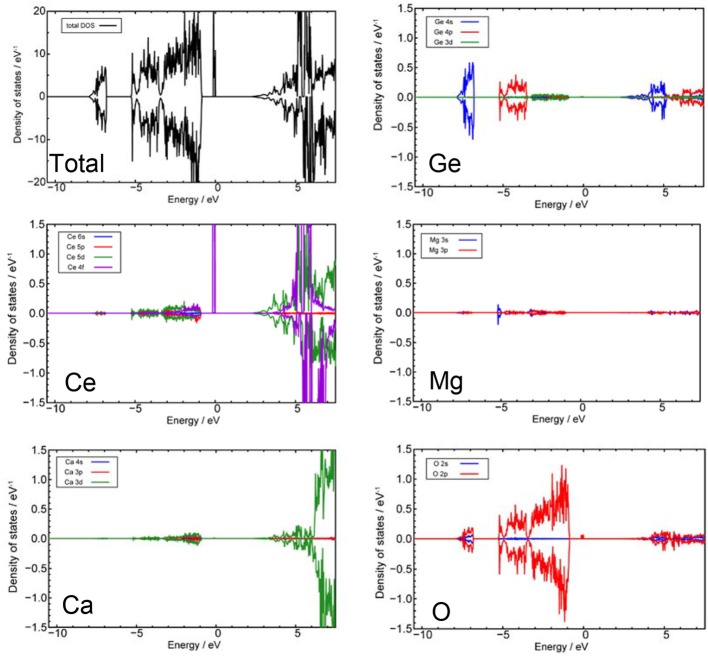
Total and projected partial density of states calculated from the model shown in Figure 6. The energy level of a valence band top is set to be 0 eV on the horizontal axes. Positive and negative values of the vertical axes indicate the DOS of up-spin and down-spin, respectively. Blue, red, green, and purple lines indicate the *s, p, d*, and *f* orbitals of each element, respectively.

From the series of total energy calculations of Ce_2_CaMg_2_Ge_3_O_12_ models, the most stable configuration that was found is shown in Figure 6. We analyzed the electronic structures of this model. Figure 7 shows total density of states (tDOS) and projected partial density of states (pDOS) of each constituent element. In Figure 7, the energy level of a valence band top is set to be 0 eV on the horizontal axes. Positive and negative values on the vertical axes indicate the DOS of up-spin and down-spin, respectively. The tDOS values show that the calculated band gap is about 2.2 eV, which is in a good agreement with the value estimated from the UV–vis absorption spectrum. It can be clearly seen that very sharp spikes of the DOS exist at the topmost energy levels of the occupied states. Such sharp DOS peaks indicate strong localization states of the electron orbitals. From the pDOS values, we can see that these peaks originate from the occupied 4f orbital of the Ce^3+^ ions. The DOS near the conduction band bottom seems to be mainly composed of an unoccupied 5d orbital of Ce^3+^ ions and a 4s orbital of the Ge^4+^ ions.

## Conclusion

We have successfully synthesized a new metastable germanate garnet, Ce_2_CaMg_2_Ge_3_O_12_, using a flux crystal growth method. Reddish-orange single crystals were grown in a reactive MgO crucible; however, the polycrystalline sample could not be prepared via a solid state reaction. Flux reactions are clearly useful for extending the garnet family to compositions that include the early lanthanide metals, especially those larger than Gd, which have been less explored. The PL intensity was so weak that it could not be confirmed visually; this is probably due to Ce^3+^-concentration quenching effects or photoionization involving a charge transfer between Ce^3+^ and Ge^4+^ (Blasse et al., 1991; Pinelli et al., 2004). Work to synthesize La or *RE* (<Ce^3+^)-doped Ce_2_CaMg_2_Ge_3_O_12_ and the substitution of Si for Ge, which would enhance PL properties, is ongoing.

## Data Availability Statement

The datasets generated for this study can be found in the Cambridge Crystallographic Data Centre (https://www.ccdc.cam.ac.uk/structures/) under the identifier 1969400.

## Author Contributions

The manuscript was written through contributions of all authors. All authors have given approval to the final version of the manuscript.

### Conflict of Interest

The authors declare that the research was conducted in the absence of any commercial or financial relationships that could be construed as a potential conflict of interest.

## Supplementary Material

CIF files of Ce_2_CaMg_2_Ge_3_O_10_ based on single crystal XRD data. Anisotropic displacement parameters for Ce_2_CaMg_2_Ge_3_O_10_. Crystallographic data obtained from the Rietveld refinement against the SXRD data. Powder XRD data collected from the products of the solid state reactions.
