# Supplementary material for: Flux Crystal Growth, Crystal Structure, and Optical Properties of New Germanate Garnet Ce2CaMg2Ge3O12
Source: Front Chem. 2020 Feb 18;8:91. doi: 10.3389/fchem.2020.00091 (PMC7039934; doi:10.3389/fchem.2020.00091)
Supplement: Supplementary file 1 [file Table_1.DOCX]

Supplementary Material


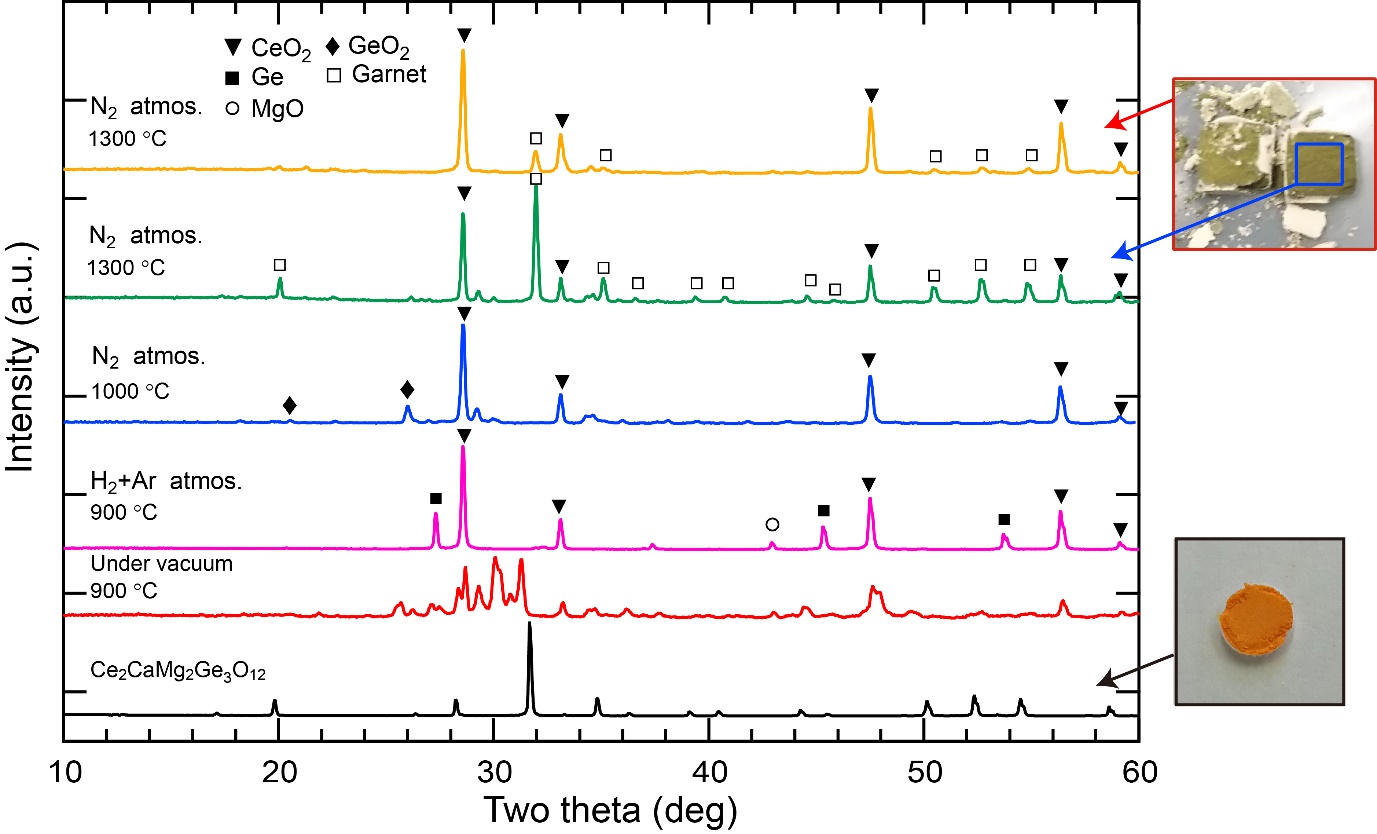


**Supplementary Figure 1.** Powder XRD patterns of the products obtained from the solid-state reactions. For comparison, the PXRD data of the powder sample of Ce_2_CaMg_2_Ge_3_O_12_ which was obtained by crushing the single crystals is plotted together. The reaction at 900 °C in an evacuated tube resulted in a complex powder pattern. GeO_2_ was reduced to Ge metal in H_2_(20%)+Ar(80%) mixture gas atmosphere. Several peaks which could be assigned to the garnet structure were observed from the product heated at 1300 °C in N_2_ gas atmosphere, but the lattice constant was smaller by 0.5% than that for Ce_2_CaMg_2_Ge_3_O_12_. The sample color was also different from the reddish-orange color of Ce_2_CaMg_2_Ge_3_O_12_.

**Supplementary Table 1.** Anisotropic displacement parameters *U_ij_* (10^2^×Å^2^) for Ce_2_CaMg_2_Ge_3_O_12_ obtained from the structure refinement using single-crystal XRD data

| Atom | *U*_11_ | *U*_22_ | *U*_33_ | *U*_12_ | *U*_13_ | *U*_23_ |
| --- | --- | --- | --- | --- | --- | --- |
| Ce/Ca | 0.63(2) | 0.791(18) | 0.791(18) | 0.05(2) | 0 | 0 |
| Mg | 0.75(5) | 0.75(5) | 0.75(5) | -0.05(8) | -0.05(8) | -0.05(8) |
| Ge | 0.72(3) | 0.63(2) | 0.63(2) | 0 | 0 | 0 |
| O | 0.52(11) | 0.78(12) | 0.75(12) | 0 | 0.16(10) | 0.12(9) |

**Supplementary Table 2.** Atomic coordinates and isotropic displacement parameters *U*_iso_ refined from SXRD data collected from Ce_2_CaMg_2_Ge_3_O_12_ at room temperature

| Atom | Site | *x* | *y* | *z* | *Occp.* | *U*_iso_ (Å^2^×10^2^) |
| --- | --- | --- | --- | --- | --- | --- |
| Ce*^a^* | 24c | 1/8 | 0 | 1/4 | 0.667 | 0.43(2) |
| Ca*^a^* | 24c | 1/8 | 0 | 1/4 | 0.333 | 0.43 |
| Mg | 16a | 0 | 0 | 0 | 1 | 0.15(4) |
| Ge | 24d | 3/8 | 0 | 1/4 | 1 | 0.56(2) |
| O | 96h | 0.0944(2) | 0.1966(2) | 0.2880(2) | 1 | 0.27(7) |

*^a^* For Ce and Ca atoms occupying the same site (24*c*), their site occupancies were fixed at 0.667 and 0.333, respectively, so that the sum of them was equal to unity. The *U*_iso_ values as well as the atomic coordinates were also constrained to the same values.
